# Effects of free maternal policies on quality and cost of care and outcomes: an integrative review

**DOI:** 10.1017/S1463423621000529

**Published:** 2021-09-15

**Authors:** Boniface Oyugi, Sally Kendall, Stephen Peckham

**Affiliations:** 1 Centre for Health Services Studies, University of Kent, Canterbury, UK; 2 The University of Nairobi, Nairobi, Kenya

**Keywords:** cost of care, free maternity policy, free delivery policy, quality of care, universal health coverage

## Abstract

**Aim:**

We conducted an integrative review of the global-free maternity (FM) policies and evaluated the quality of care (QoC) and cost and cost implications to provide lessons for universal health coverage (UHC).

**Methodology:**

Using integrative review methods proposed by Whittemore and Knafl (2005), we searched through EBSCO Host, ArticleFirst, Cochrane Central Registry of Controlled Trials, Emerald Insight, JSTOR, PubMed, Springer Link, Electronic collections online, and Google Scholar databases guided by the preferred reporting item for systematic review and meta-analysis protocol (PRISMA) guideline. Only empirical studies that described FM policies with components of quality and cost were included. There were 43 papers included, and the data were analysed thematically.

**Results:**

Forty-three studies that met the criteria were all from developing countries and had implemented different approaches of FM policy. Review findings demonstrated that some of the quality issues hindering the policies were poor management of complications, worsened referral systems, overburdening of staff because of increased utilisation, lack of transport, and low supply of stock. There were some quality improvements on monitoring vital signs by nurses and some procedures met the recommended standards. Equally, mothers still bear the burden of some costs such as the purchase of drugs, transport, informal payments despite policies being ‘free’.

**Conclusions:**

FM policies can reduce the financial burden on the households if well implemented and sustainably funded. Besides, they may also contribute to a decline in inequity between the rich and poor though not independently. In order to achieve the SDG goal of UHC by 2030, there is a need to promote awareness of the policy to the poor and disadvantaged women in rural areas to help narrow the inequality gap on utilisation and provide a sustainable form of transport through collaboration with partners to help reduce impoverishment of households. Also, there is a need to address elements such as cultural barriers and the role of traditional birth attendants which hinder women from seeking skilled care even when they are freely available.

## Background

While maternal deaths have reduced by nearly 50% since 1990, the Sustainable Development Goals (SDGs) aim to further decrease the maternal mortality ratio (MMR) to less than 70 per 100 000 live births by 2030 (United Nations, 2017b). Many countries have made relatively little progress so far. A recent systematic analysis of maternal mortalities in 181 countries from 1980 to 2008 showed that while there was progress in achieving reduced MMR, only 23 countries including China, Egypt, Bolivia, and Ecuador were on track to achieve a considerable decrease of 75% (Hogan *et al.*, 2010). From the global estimates of 2017, there are nearly 295 000 maternal mortalities that happen globally, mainly from complications related to pregnancy and childbirth (World Health Organisation, 2019). Low- and middle-income countries (LMICs) account for the majority of the high burden of the mortalities (Carrera, 2007). For instance, MMR in Benin is estimated at 405 deaths in 100 000 live births (Dossou *et al.*, 2018), 575 in Nigeria (Oyeneyin *et al.*, 2017), and as high as 1360 in Sierra Leone (Koroma *et al.*, 2017). Poor use of lifesaving maternal services and family planning services contributes to the high MMR in developing countries which is 14 times higher than the developed countries (United Nations, 2017b).

Women are increasingly forming the backbone of many families as breadwinners (Amu, 2005), and their death can push the whole family to penury. Women’s increased risks of dying in pregnancy are primarily due to preventable causes (Koroma *et al.*, 2017) such as haemorrhage, toxaemia, unsafe abortion, and obstructed labour (Hulton *et al.*, 2000). In Sub-Saharan Africa (SSA), such deaths are mainly caused by lack of timely access to skilled delivery caused by the delayed decision by individuals and family to seek care, delay in getting to the health facility, and delay in the provision of adequate care by the facility. Gabrysch and Campbell (2009) identified and grouped 20 determinants that affect skilled institutional deliveries into four themes as socio-cultural factors, the perceived benefits and needs of skilled birth attendance, economic accessibility, and physical accessibility. Besides, they suggested the role of quality of care (QoC) which is, in most instances, not captured in household surveys, the role of distance, and the ability to pay. While increasing service availability is perceived as imperative, it does not always increase the use of the service (Hulton *et al.*, 2000). Both the perceived and actual quality of maternal and neonatal healthcare are imperative because they influence the decision to seek healthcare.

On the other hand, high out-of-pocket (OOP) expenditure is increasingly forcing households into poverty (Xu *et al.*, 2003). Globally, there are approximately 150 million who experience health-related catastrophic expenditure, of which 100 million fall into poverty (Xu *et al.*, 2007). This health catastrophic expenditure is both in low- and high-income countries, but over 90% of people who suffer the most are in LMIC (Xu *et al.*, 2003). An analysis of the nationally representative survey in Malaysia, Sri-Lanka, Indonesia, and Thailand showed that of the 2.7% of the population under survey, approximately 78 million remained with less than one-dollar-a-day after paying for health care and that the exemption policies, particularly for the more impoverished people, was an important strategy to mitigate such payments that could lead to impoverishment (Van Doorslaer *et al.*, 2006).

Many countries have implemented financial incentives to address the element of QoC and outcome, catastrophic cost, and equitable utilisation of maternal healthcare services (Stanton *et al.*, 2013) and to achieve universal health coverage (UHC). One such incentive is the removal of user fees for primary health care (PHC) which also covers maternal healthcare and is aimed at reducing pregnancy and childbirth-related morbidities and mortalities. One study that mapped countries that implemented free policies showed that of the 49 countries selected for mapping, more than half (28) were exclusively focusing on free delivery care or were being implemented together with other curative services (Witter, 2010).

This integrative review (IR) limits itself to analysing the QoC and outcome, and the cost implications of free maternity (FM)/delivery policies to provide lessons for UHC. The review complements the findings on the utilisation of services under FM policies – links with existing literature on utilisation because many prior analyses have mainly focused on comparing the changes in utilisation of services before and after the implementation of the free policies (Ridde, 2003; Masiye *et al.*, 2008; Nabyonga-Orem *et al.*, 2008). Some studies that have gone beyond evaluating the changes in utilisation before and after the free policy have mainly focussed on the preventative and curative care themes (Lagarde *et al.*, 2012) and not maternal health services. Therefore, this review focuses on the free maternal or delivery healthcare policies as implemented globally.

### Purpose of the integrative review study

The review answers the following questions:

1. What are the approaches to implementation of FM policy?
2. What is the quality (care and outcomes) and the cost implications about policy and practice that legislate for the free global maternity care?
3. What lessons can we learn from the global FM policy to support the achievement of UHC?

## Methods

### Study design

The study utilised the integrative literature review, which allows for the synthesis of several streams of literature (Whittemore and Knafl, 2005; Yorks, 2008). The method was useful for reviewing, critiquing, and synthesising evidence from research in an integrative way that allowed new perspectives and frameworks to be drawn (Christmals and Gross, 2017; Rosa *et al.*, 2017). In particular, the study included a wide range of literature from several fields of study which were analysed through a multidisciplinary approach. Also, the study focused on peer-reviewed literature, models, frameworks, policy documents on free maternal/delivery policies that reported on elements of quality and cost of care and outcomes.

### Search methods

We searched for articles in databases and sources, as shown in Table 1. All articles that met the criteria were included irrespective of the year of publication. We then reviewed the bibliographies of all studies identified after which we reached the saturation point and provided a comprehensive list that was validated by SK. Boolean operators (OR and AND) were used to limit and expand the search as appropriate. We had four sets of search terms (Table 2) adapted and modified from Ridde and Morestin (2010) and Ridde *et al.* (2012c) which were combined using OR within each set and AND linking different sets. All searches were imported to Endnote library and pooled, after which duplicates were removed.

**Table 1.** Source of evidence

*e-journal services and electronic databases*	Applied social sciences index and abstracts, EBSCO Host (Academic search complete, Cumulative Index to Nursing and Allied health Literature (CINAHL), Econ Lit, MEDLINE, and PsycINFO), ArticleFirst, Cochrane Central Registry of Controlled Trials (Cochrane Library, Wiley Online Library), Emerald Insight, JSTOR, PubMed, Springer Link (Springer Reference), Google scholar, and Electronic Collections Online.
*Health Sources*	International Bibliography of the Social Sciences, SAGE journals Online, Nursing/Academic Edition, Latin-American and Caribbean Centre on Health Sciences Information (LILACS), African Journals Online, SCOPUS, Science Direct, Web of Science (Web of Knowledge & Science Citation Index), OVID (Social Policy and Practice).
*Websites on health financing*	World Health Organisation, The World Bank, Mednar, Intute, Nexis UK, Qual page, Scirus, WorldWideScience.org, and Eldis
*Grey Literature*	SIGLE database

**Table 2.** Search words

(“User fee*” OR “user charg*” OR “cost shar*” OR “cost recover*” OR “User Fee* Policy” OR “User Policy”)) AND
(“Free polic*” OR “Free policy” OR “Free Health*” OR “Free Car*” OR “free care” OR “Discontin*” OR “Abol*” OR “exempt*” OR “waiv*” OR “Remov*” OR “end*” OR “Policy Chang*” OR “Chang*” OR “implement*” OR “Policy implement*”)) AND
(“matern*” OR “deliver*” OR “Mother car*” OR “baby car*” OR “infant car*” OR “matern* car*” OR “deliver* car*” OR “giv* birth” OR “labour” OR labor OR mother*” OR “Childbirth*” OR “birth” OR “parturit*” OR “accouch*”)) AND
(“Quality” OR “quality car*” OR “health care quality” OR “quality outcome*” OR “cost*” OR “Expenditure” OR “Economic*” OR “Financ*” OR “effective Cover*” OR “Universal Care” OR “Universal Health Cover*” OR “Universal Health”

### Selection criteria

**Figure 1.**
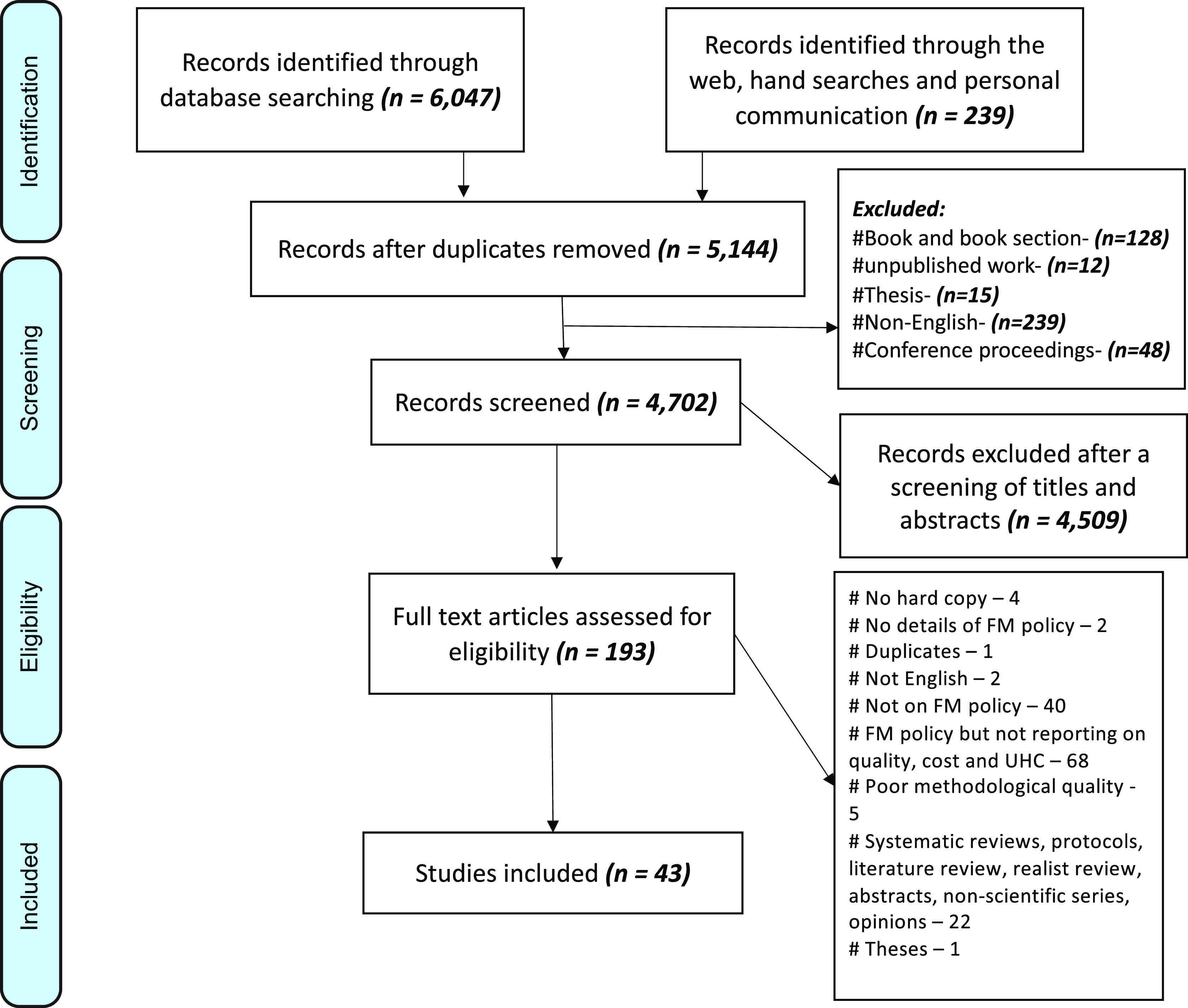
Flow chart of the selection of studies for review

The review was limited to studies conducted in English that reported the concepts of implementation, quality and cost of care and outcomes of FM policies, and lessons for UHC. We included all study types, and the final review included experimental studies, quantitative, qualitative, and mixed-method studies done in single or multiple countries. Some of the excluded studies had no relevance to healthcare, had a poor methodological approach, and were talking about free maternity services (FMS) but not the quality or cost of the free maternal healthcare policy. First, all titles were screened for eligibility. Second, studies that met eligibility had their abstract further screened for eligibility after which full texts were screened for those studies that meet the eligibility criteria. All articles were included irrespective of the date. Figure 1 shows the search outcomes.

### Quality appraisals

In this review, we did not do meta-analysis because there was much heterogeneity in the studies. We applied a mix of validated critical quality appraisal techniques (Wechkunanukul *et al.*, 2017). First, we applied the checklist for writing an integrative review as stipulated by Toracco (2005) and utilised the Preferred Reporting Item for Systematic Reviews and Meta-Analysis (PRISMA) checklist for reporting systematic reviews (Moher *et al.*, 2015) (Figure 1). For the quality appraisal of the individual study designs, we applied the Critical Appraisal Skills Programme (CASP) tools (Critical Appraisal Skills Programme, 2017a; 2017b; 2017c; 2017d; 2017e; 2017f, 2017g; 2017h). All the articles were, through a sampling technique, evaluated by a second researcher at the last stage, and where there was disagreement, the resolution was made through consultation.

### Data abstraction, analysis and evaluation

There were five steps applied in this review: data reduction, display, comparison, concluding, and verification (Torraco, 2005; Whittemore and Knafl, 2005; Wechkunanukul *et al.*, 2017). Data reduction was classified by the subgroup classification of studies based on research design. Data were abstracted and entered into Microsoft Excel®, from where the synthesis of the text was done through thematic analysis using variables of interest and conclusions are drawn.

### Definitions and frameworks

Quality cannot be measured by itself (Kelley and Hurst, 2006) and Donabedian broadly classifies it as structure, process, and outcome dimensions (Donabedian, 1988; Donabedian, 1990) which can be identified, measured, and attributed to healthcare. Structure indicators represent pointers which are inputs to or characteristics of health; process indicators represent gauges to either appropriate or inappropriate care in a targeted population which are “consistent with current professional knowledge”; outcome indicators are the measures of both improved or deteriorated health and is attributed to medical care (Kelley and Hurst, 2006). In this review, we broadly defined QoC using the quality of maternal and newborn healthcare framework as proposed by the World Health Organisation (WHO) (2016). There are eight domains of QoC in the framework that targets mothers and their newborns in the health system (hospitals), making it likely to achieve the desired individual and facility-level outcome. The approach gives two quality improvement standards: provision and experience of care. Provision of care supports evidence-based practices for routine care and management of complications, actionable information system, and functional referral system, while the experience of care supports effective communication, respect and preservation of dignity, and emotional support. There are two cross-cutting areas of QoC, namely: competent, motivated human resources and essential physical resources available.

**Figure 2.**
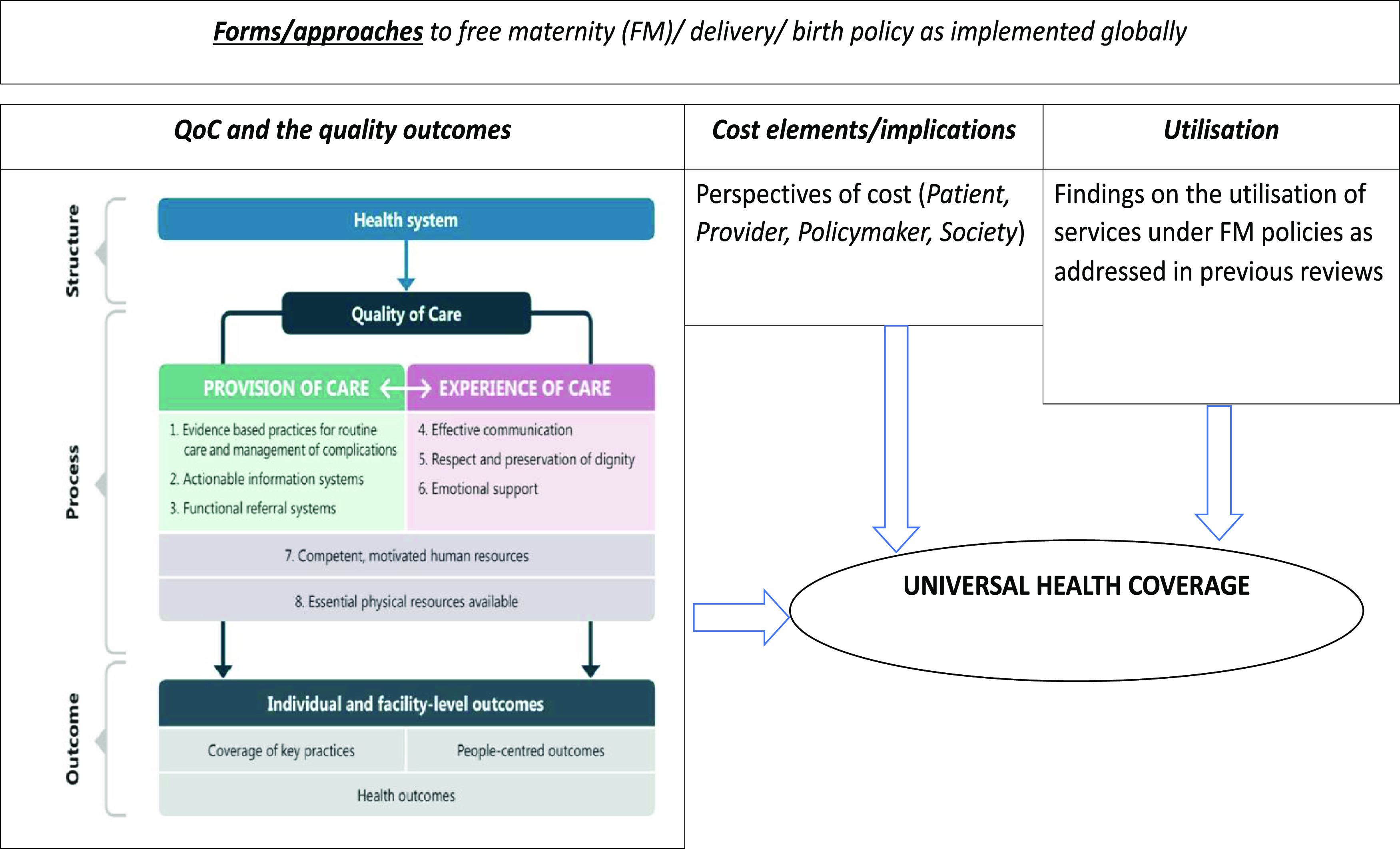
Analysis framework (Source: Review of literature and WHO framework for the quality of maternal and newborn health care)

On the other hand, increased cost of care is repeatedly attributed to the cause of reduced use of services (Mekonnen and Mekonnen, 2003). In this review, costs elements are defined thematically from the perspective of the patients, provider, and the policymakers (Figure 2).

## Results

### Description of the studies

*Additional file 1* shows an overview of all the papers included in the review. The IR identified 6047 articles published without date restriction until December 2017 and an addition of 239 identified through the web, hand searches, and personal communications. After filtering for duplicates, 5,144 articles were considered for review. The articles then had their titles and abstracts screened for eligibility for which 43 articles met all the criteria and thus included in the final stage of data abstraction (*Additional file 1*).

The quality scores of the individual studies are reported in *Additional file 1*. The quality appraisal of 21 studies included in the review was rated as high because they reported sufficient details about the FM policy and highlighted robust methodologies with findings according to the appraisal tools used (Witter *et al.*, 2007; Masiye *et al.*, 2010; Witter *et al.*, 2010; Nimpagaritse and Bertone, 2011; Steinhardt *et al.*, 2011; Witter *et al.*, 2011; Ameur *et al.*, 2012; Ridde *et al.*, 2012b; Witter *et al.*, 2012; Arsenault *et al.*, 2013; Ridde *et al.*, 2013; Ganle *et al.*, 2014; Delamou *et al.*, 2015; Ravit *et al.*, 2015; Ridde *et al.*, 2015; Boukhalfa *et al.*, 2016; Ganaba *et al.*, 2016; Witter *et al.*, 2016; Chankham *et al.*, 2017; Ensor *et al.*, 2017; Witter *et al.*, 2017); 13 studies were rated medium (Asante *et al.*, 2007; Bosu *et al.*, 2007; Kruk *et al.*, 2008; Nabyonga-Orem *et al.*, 2008; Ridde and Diarra, 2009; Ridde *et al.*, 2012a; The World Bank, 2013; Chama-Chiliba and Koch, 2014; Philibert *et al.*, 2014; Chama-Chiliba and Koch, 2016; Lange *et al.*, 2016; Dalinjong *et al.*, 2017; Koroma *et al.*, 2017); and six studies were rated low because they either did not report their study design or their description of the method section was not sufficient but captured enough information on the FM policy (Nahar and Costello, 1998; Khan, 2005; Kenya Ministry of Health, 2015; Sidze *et al.*, 2015; Vallières *et al.*, 2016; Edu *et al.*, 2017). One study did not address the criteria for methodological quality (Luwei *et al.*, 2011).

On study designs, two studies did not outrightly indicate the study design but indicated their study methods (Luwei *et al.*, 2011; Sidze *et al.*, 2015); eleven utilised a cross-sectional design (Nahar and Costello, 1998; Khan, 2005; Asante *et al.*, 2007; Bennis and De Brouwere, 2012; Ridde *et al.*, 2012a; The World Bank, 2013; Ridde *et al.*, 2015; Boukhalfa *et al.*, 2016; Vallières *et al.*, 2016; Chankham *et al.*, 2017; Koroma *et al.*, 2017); four case control designs (Ameur *et al.*, 2012; Arsenault *et al.*, 2013; Philibert *et al.*, 2014; Ravit *et al.*, 2015); one cohort study (Nabyonga-Orem *et al.*, 2008), and seven case studies (Masiye *et al.*, 2010; Nimpagaritse and Bertone, 2011; Ridde *et al.*, 2012b; Philibert *et al.*, 2014; Ganaba *et al.*, 2016; Witter *et al.*, 2016; Witter *et al.*, 2017). Other study designs indicated in the papers were: one interrupted time series design (Chama-Chiliba and Koch, 2016); 3 quasi-experimental design studies (Chama-Chiliba and Koch, 2014; Philibert *et al.*, 2014; Ensor *et al.*, 2017); three studies had components of before and after intervention study design (Bosu *et al.*, 2007; Masiye *et al.*, 2010; Witter *et al.*, 2011); one descriptive convergent parallel mixed method design (Dalinjong *et al.*, 2017); and one mixed method sequential explanatory design (Ridde *et al.*, 2013). The other studies were evaluations studies that applied varied evaluation approaches (and not one specific design). For instance, three studies used realist approaches (Ganle *et al.*, 2014; Witter *et al.*, 2016; Witter *et al.*, 2017); one descriptive and analytical implementation evaluation (Ridde *et al.*, 2013) and another policy implementation evaluation (Witter *et al.*, 2012); two process evaluations with varied designs (Ridde and Diarra, 2009; Witter *et al.*, 2013); one monitoring and evaluation using mixed methods (Kenya Ministry of Health, 2015); and one monitoring and evaluation using a before and after study design (Witter *et al.*, 2011). Others are one outcome evaluation (Steinhardt *et al.*, 2011), one policy baseline evaluation (Witter *et al.*, 2007), and another policy evaluation (Witter *et al.*, 2010). See *Additional file 1*.

A closer analysis of the articles revealed that three articles were multi-country studies, with two of them reporting on both low-income countries (LIC) and LMIC countries (Witter *et al.*, 2016; Witter *et al.*, 2017), one being a transversal analysis of entirely LICs (Ridde *et al.*, 2012b), 21 evaluated singular counties that were LICs (Kruk *et al.*, 2008; Nabyonga-Orem *et al.*, 2008; Ridde and Diarra, 2009; Witter *et al.*, 2010; Luwei *et al.*, 2011; Nimpagaritse and Bertone, 2011; Steinhardt *et al.*, 2011; Witter *et al.*, 2011; Ameur *et al.*, 2012; Ridde *et al.*, 2012a; Arsenault *et al.*, 2013; Ridde *et al.*, 2013; Philibert *et al.*, 2014; Delamou *et al.*, 2015; Ravit *et al.*, 2015; Ridde *et al.*, 2015; Ganaba *et al.*, 2016; Lange *et al.*, 2016; Vallières *et al.*, 2016; Ensor *et al.*, 2017; Koroma *et al.*, 2017), and 19 were in LMICs (Nahar and Costello, 1998; Khan, 2005; Asante *et al.*, 2007; Bosu *et al.*, 2007; Witter *et al.*, 2007; Masiye *et al.*, 2010; Bennis and De Brouwere, 2012; Witter *et al.*, 2012; The World Bank, 2013; Witter *et al.*, 2013; Chama-Chiliba and Koch, 2014; Ganle *et al.*, 2014; Kenya Ministry of Health, 2015; Sidze *et al.*, 2015; Boukhalfa *et al.*, 2016; Chama-Chiliba and Koch, 2016l Chankham *et al.*, 2017; Dalinjong *et al.*, 2017; Edu *et al.*, 2017). No work from high income country (HIC) met the criteria. See *Additional file 1.*

### Forms of free maternity policy by different countries

**Figure 3.**
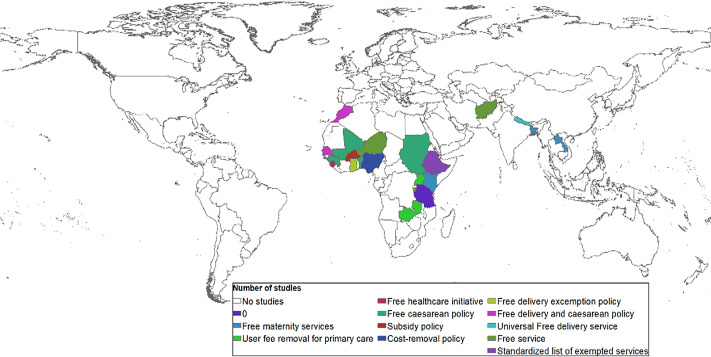
Forms of policy implementation of free maternity policy (Source: authors mapped from review of literature)

The review found different forms of the FM policies, which are mainly implemented in LICs and LMICs (Figure 3). The FM policy processes of several countries that were reviewed required a significant amount of resources, political processes, and social institutions to institutionalise, maintain, or shape their structures (Lightman and Lightman, 2017). In some countries, the processes were well planned to use the steps of the stage heuristic model, others required a window of opportunity to implement, and some were born out of need.

### Quality of maternal care

The QoC and outcome reported in this section utilised the WHO proposed framework (World Health Organisation, 2016) from the perspective of the managers, service providers, and users in two aspects: provision and experience of care.

#### Provision of care

The findings on provision of care are in three main areas: evidence-based practices for routine care and management of complications, actionable information systems, and functional referral system.

##### Evidence-based practices for routine care and management of complications

Eight studies reviewed had positive, evidence-based practices for routine care and management of complication with the FM Policy (Bosu *et al.*, 2007; Steinhardt *et al.*, 2011; Delamou *et al.*, 2015; Kenya Ministry of Health, 2015; Sidze *et al.*, 2015; Vallières *et al.*, 2016; Witter *et al.*, 2016; Edu *et al.*, 2017; Koroma *et al.*, 2017), seven studies had contrary evidence (Witter *et al.*, 2007; Witter *et al.*, 2012; Sidze *et al.*, 2015; Ganaba *et al.*, 2016; Lange *et al.*, 2016; Witter *et al.*, 2016; Koroma *et al.*, 2017), while three showed no change after implementation of FM policy (Luwei *et al.*, 2011; Kenya Ministry of Health, 2015; Chama-Chiliba and Koch, 2016).

For instance, the authors linked the policies to improved perception of QoC index by hospitals (Steinhardt *et al.*, 2011) and mothers (Koroma *et al.*, 2017). FM policies have been linked with an increase in the number of ANC visits (Kenya Ministry of Health, 2015), improved screening for vital signs such as weight, foetal movements (Koroma *et al.*, 2017), measurements of BP (Sidze *et al.*, 2015; Koroma *et al.*, 2017), screening of urine and blood, and maternal immunisation for tetanus at ANC (Sidze *et al.*, 2015). Equally, they were linked with increased immunisation of children (Vallières *et al.*, 2016) and more mothers being counselled on breastfeeding and pregnancy-related complication during ANC (Sidze *et al.*, 2015). Maternal complications were either identified early, referred and hospitalised as appropriate (Delamou *et al.*, 2015; Edu *et al.*, 2017), or declined (Kenya Ministry of Health, 2015), leading to reduced maternal deaths (Bosu *et al.*, 2007; Kenya Ministry of Health, 2015). Importantly, the policies are associated with a reduction in the stillbirths, underweight babies, and preterm babies (Kenya Ministry of Health, 2015). Significantly, there was a reduced transmission of HIV at birth from mother to child (Kenya Ministry of Health, 2015), and some hospitals had the best technical quality care measured by reduced delays, low omission scores, and low fatality rates for adverse complications (Witter *et al.*, 2016).

In some policies, the HCWs were poorly using the partograph (Witter *et al.*, 2007), others were not adhering to treatment guidelines and procedures (Witter *et al.*, 2012) or were using treatment methods perceived as not sterile (Lange *et al.*, 2016). Others had critical omissions in routine procedures (Witter *et al.*, 2016) and even concentrated more on complicated and surgical deliveries than vaginal deliveries (Ganaba *et al.*, 2016). From a hospital perspective, facilities were unable to manage emergencies such as infections, pre-eclampsia, haemorrhage, anaemia, breech birth because they lacked essential lifesaving skills and emergency equipment (Koroma *et al.*, 2017). Others showed increased maternal complications such as obstructed labour (Sidze *et al.*, 2015) and increased burden of maternal and neonatal near misses (Witter *et al.*, 2016).

Luwei *et al.* (2011) revealed that there was little significant difference between facilities providing free care and facilities charging a fee on the standard parameters of quality such as the use of a partograph to monitor labour, availability of oxytocin, managing direct obstetric complications, and the availability of new-born resuscitation procedures. There was limited evidence of better patient safety measures and of assuring/monitoring adherence to expected care standards (Kenya Ministry of Health, 2015). In Zambia, a before and after analysis of the free primary care demonstrated that there was little or insignificant difference in uptake of ANC despite the likelihood of good quality of ANC influencing the use of a health facility for delivery (Chama-Chiliba and Koch, 2016).

##### Actionable information systems

Only two studies reported on information system (Ridde and Diarra, 2009; Chankham *et al.*, 2017). Ridde and Diarra (2009) in their process evaluation of FM services in Niger highlighted that the new policy introduced a parallel operating system to the already existing system which meant that the HCWs mainly had to provide care to different groups of patients registered in the free program and the old program resulting to increased workload. Chankham *et al.* (2017) noted that the provision of information at the health facilities enhanced satisfaction with the quality-of-service provision.

##### Functional referral

Nine studies reviewed reported on the element of the referral system relating to FM policies (Witter *et al.*, 2007; Ridde and Diarra, 2009; Nimpagaritse and Bertone, 2011; Ridde *et al.*, 2012b; The World Bank, 2013; Ganle *et al.*, 2014, Delamou *et al.*, 2015; Sidze *et al.*, 2015; Edu *et al.*, 2017). Studies showed that FM policies resulted in proper referral where patients were referred from lower-level to higher-level facilities (Sidze *et al.*, 2015; Edu *et al.*, 2017) which in turn resulted in early detection of and reduced complications (Edu *et al.*, 2017). Reimbursement strategies played a role in referral. For instance, hospital reimbursement strategies that were pegged on successful referral helped to improve quality outcomes (Witter *et al.*, 2007). However, reimbursing hospitals based on the number and type of deliveries conducted resulted in some unintended consequences. Some unintended consequence included lower facilities not referring patients for fear of losing revenue as is the case of Ghana (Witter *et al.*, 2007), or HCWs choosing caesarean section over normal birth even if unwarranted for higher revenues as the case in Laos PDR (The World Bank, 2013).

With regard to the implementation of the FM policies, it was evidenced that there was uncoordinated and unreimbursed referral strategies (Witter *et al.*, 2007) and some faced lack of critical staff to handle referred emergencies (Ganle *et al.*, 2014). In some counties such as Guinea, there was a significant decrease in unmet obstetric need after implementation of the policy as hospitals were incentivised to handle complications hence reduce referral (Delamou *et al.*, 2015). By including the transport component to the FM policy, there was improved satisfaction outcomes and satisfaction with the services (Ridde *et al.*, 2012b). However, specific gaps in referral after policy implementation such as inadequate or lack of follow-up to ensure the evacuated mothers received care as intended (Ridde and Diarra, 2009) or in cases where implementation is done rapidly, there was a disruption of referral system (Nimpagaritse and Bertone, 2011).

#### Experience of care

The findings on the experience of care highlight three main areas of quality: cognition and effective communication, respect and preservation of dignity, and emotional support.

##### Cognition and effective communication

Three studies highlighted how cognition and effective communication influenced perception or technical elements of quality (Lange *et al.*, 2016; Witter *et al.*, 2016; Edu *et al.*, 2017). How the HCWs communicated to the mothers determined their perception of confidence in handling complications (Edu *et al.*, 2017). The studies showed that the nurses had not well-informed mothers about delivery procedures and that there was lack of proper reason for the procedure in cases of emergency (Lange *et al.*, 2016). Equally, poor communication between HCWs and mothers influenced the lack of informed consent for surgery and poor bedside manners (Witter *et al.*, 2016). Besides, nurses took time to decide between having to do a CS or normal delivery with inconsistencies in scheduling a CS because of the surgeon’s unavailability (Lange *et al.*, 2016). Mothers perceived some negligence by HCWs who provided inadequate care and support of the perineum during vaginal delivery (Lange *et al.*, 2016).

##### Respect and preservation of dignity

Eight studies reviewed highlighted components of respect and preservation of dignity (Witter *et al.*, 2007; Nabyonga-Orem *et al.*, 2008; Masiye *et al.*, 2010; Ganle *et al.*, 2014; Philibert *et al.*, 2014; Kenya Ministry of Health, 2015; Witter *et al.*, 2016; Edu *et al.*, 2017). Some highlighted respect concerns were harsh treatment from some HCWs (Edu *et al.*, 2017), negative attitude towards the women (Witter *et al.*, 2007; Ganle *et al.*, 2014), staff being too few, rude, and not available when required (Nabyonga-Orem *et al.*, 2008), and poor interpersonal relationship between the mothers and HCWs (Witter *et al.*, 2016). In relation to this, the women distrusted the knowledge, practices, skills, and competence of the maternal healthcare providers mothers because they were chided and scolded for not attending ANC early and a high throughput of patients made it difficult to maintain privacy for the mothers during procedures (Ganle *et al.*, 2014).

However, some studies showed that HCWs had shown a kind attitude (Edu *et al.*, 2017) and excellent interpersonal skills (Kenya Ministry of Health, 2015) towards pregnant mothers. Others had little indication to propose that staff courtesy had deteriorated after the policy change (Masiye *et al.*, 2010), or that the provider–patient interaction, nursing care, and the delivery environment had changed (Philibert *et al.*, 2014).

##### Emotional support

Only one study reviewed showed captured elements of emotional support. The study showed that mothers who were experiencing difficulty with breastfeeding had received emotional support from their HCWs, who provided adequate breastfeeding counselling (Sidze *et al.*, 2015).

#### The aspect that cut across both provision and experience of care

Competent, motivated human resource and essential physical resources available are two QoC areas that cut across both provision and experience of care and that do not fit across the eight categories.

##### Competent, motivated human resource

Sixteen studies reviewed highlighted the components of competency and motivation of HCWs following the implementation of FM policies (Bosu *et al.*, 2007; Witter *et al.*, 2007; Nabyonga-Orem *et al.*, 2008; Masiye *et al.*, 2010; Nimpagaritse and Bertone, 2011; Witter *et al.*, 2011; Witter *et al.*, 2012; Ganle *et al.*, 2014; Kenya Ministry of Health, 2015; Sidze *et al.*, 2015; Lange *et al.*, 2016; Vallières *et al.*, 2016; Witter *et al.*, 2016; Chankham *et al.*, 2017; Koroma *et al.*, 2017; Witter *et al.*, 2017). Factors that were highlighted as determinants of competency and motivation were functionality of infrastructure (Witter *et al.*, 2012), the type of working conditions (Vallières *et al.*, 2016), presence or absence of incentives from reimbursements for services (Witter *et al.*, 2012; Vallières *et al.*, 2016; Koroma *et al.*, 2017), transparency in the use of reimbursements by in charges (Masiye *et al.*, 2010), timeliness of reimbursements (Kenya Ministry of Health, 2015), weak guidance on the use of reimbursements (Kenya Ministry of Health, 2015), flexibility in the use of reimbursement to reward HCWs by hospitals (Witter *et al.*, 2011; Lange *et al.*, 2016), and changes in workload following free policies(Witter *et al.*, 2007; Nimpagaritse and Bertone, 2011; Witter *et al.*, 2011; Witter *et al.*, 2012; Ganle *et al.*, 2014; Kenya Ministry of Health, 2015; Lange *et al.*, 2016; Vallières *et al.*, 2016; Witter *et al.*, 2016; Koroma *et al.*, 2017; Witter *et al.*, 2017).

The factors highlighted above resulted in increased corruption by the HCWs as a result of the loss of incentive that came with user fees (Witter *et al.*, 2012; Lange *et al.*, 2016) or poor pay (Lange *et al.*, 2016), difficulty in recruitment of HCWs because of poor working conditions (Vallières *et al.*, 2016), unequal distribution of HCWs because of the functionality of infrastructure (Witter *et al.*, 2012). In some cases where workload increased, there was reduced productivity by the HCW (Witter *et al.*, 2016) which saw them spend less time with mothers as a way of coping with the higher numbers at the clinic (Edu *et al.*, 2017). Though, in others, the policy resulted in better birth outcomes and more deliveries due to HCWs working longer hours (Bosu *et al.*, 2007; Witter *et al.*, 2017) which Witter *et al.* (2017) called an efficiency gain. Some HCWs perceived workload as acceptable (Ganaba *et al.*, 2016) or reasonable (Witter *et al.*, 2017) since nurses took an equal amount of time with the patients like before the policy (Masiye *et al.*, 2010). Despite the workload changes, often the mothers and HCWs perceived either increased waiting times resulting from the policies (Witter *et al.*, 2012; Ganle *et al.*, 2014; Sidze *et al.*, 2015; Witter *et al.*, 2016) or waiting times that remained the same (Masiye *et al.*, 2010; Chankham *et al.*, 2017) but were highly satisfied with the staff behaviour and because of the belief that they were dedicated and working beyond their abilities to cater to the increase in the workload (Nabyonga-Orem *et al.*, 2008; Witter *et al.*, 2012).

##### Essential physical resources available

Fifteen studies reviewed showed mixed findings on physical resources, medication, and equipment (Nabyonga-Orem *et al.*, 2008; Ridde and Diarra, 2009; Masiye *et al.*, 2010; Nimpagaritse and Bertone, 2011; Steinhardt *et al.*, 2011; Witter *et al.*, 2011; Ridde *et al.*, 2012b; Chama-Chiliba and Koch, 2014; Kenya Ministry of Health, 2015; Sidze *et al.*, 2015; Boukhalfa *et al.*, 2016; Ganaba *et al.*, 2016; Witter *et al.*, 2016; Chankham *et al.*, 2017; Edu *et al.*, 2017). Some of the components of physical resources that were perceived positively by both the HCWs and patients after the FM policy implementation were improved physical condition and cleanliness of hospitals (Masiye *et al.*, 2010) and the health facility environment (Chankham *et al.*, 2017). In other aspects, availability of amenities such as water and toilets in the majority of the health facilities (Kenya Ministry of Health, 2015), unrestricted access to latrines, and more use of treated water (Witter *et al.*, 2016) were perceived positively. However, two studies reported that there was lack of or unstable electricity coupled with a lack of or inadequate water facilities in the hospitals after the free policy (Nabyonga-Orem *et al.*, 2008; Edu *et al.*, 2017), and one showed that there was no consistent evidence of an increase in investments on things such as infrastructure (Kenya Ministry of Health, 2015).

In terms of medication and equipment, there was a perceived readiness of medicine and medical equipment after the policy implementation (Chankham *et al.*, 2017), increase in drug availability (Nabyonga-Orem *et al.*, 2008; Ridde and Diarra, 2009; Masiye *et al.*, 2010; Chama-Chiliba and Koch, 2014), and adequate and well-organised supply of drugs (Ridde and Diarra, 2009). In cases where the drugs were not available, HCWs in Uganda sent the patients to buy them from the private clinics and drug shops (Nabyonga-Orem *et al.*, 2008). Also, in Niger, HCWs were even educated on using essential generic drugs and new treatment protocols, especially for malaria (Ridde and Diarra, 2009). Eight studies highlighted increased shortages of drugs after the implementation of the policy (Nabyonga-Orem *et al.*, 2008; Nimpagaritse and Bertone, 2011; Witter *et al.*, 2011; Ridde *et al.*, 2012b; Sidze *et al.*, 2015; Boukhalfa *et al.*, 2016; Ganaba *et al.*, 2016; Edu *et al.*, 2017) in addition to constrained equipment and other consumables (Nabyonga-Orem *et al.*, 2008; Witter *et al.*, 2011; Ganaba *et al.*, 2016). Despite the shortages, the interviewed HCWs painted a rosy picture of the drug situation in Morocco (Boukhalfa *et al.*, 2016) and Nepal (Witter *et al.*, 2011). One study showed that there was no negative impact on the availability of drugs despite increased care-seeking behaviour by the patients after removal of fees (Steinhardt *et al.*, 2011).

##### Other quality elements

Four studies highlighted the roles of traditional birth attendants (TBA) as an influencer or a hinder to the achievement of QoC (Chama-Chiliba and Koch, 2014; Ganle *et al.*, 2014; Vallières *et al.*, 2016; Edu *et al.*, 2017). Two studies showed that the choice to be attended to by TBA, rather than skilled personnel in the health facility, particularly in the rural areas could not be adjusted by the changes in the cost of delivery (Chama-Chiliba and Koch, 2014; Vallières *et al.*, 2016). The negative experiences of delivery under the new policy were pushing the mothers to TBAs (Ganle *et al.*, 2014). One study showed that some mothers chose to deliver in health centres rather than through TBAs because of the poor QoC that was received in a previous pregnancy conducted by TBAs (Edu *et al.*, 2017).

### Cost elements of free maternal care

This section reports on the elements of cost of maternal care from the perspective of the managers, service providers, and users through thematic analysis.

#### Out-of-pocket (OOP) expenditures

Twenty-three of the reviewed studies showed that households in different countries still bear the burden of OOP expenditure despite the implementation of FM policies (Nahar and Costello, 1998; Khan, 2005; Kruk *et al.*, 2008; Masiye *et al.*, 2010; Witter *et al.*, 2010; Luwei *et al.*, 2011; Nimpagaritse and Bertone, 2011; Witter *et al.*, 2011; Ameur *et al.*, 2012; Bennis and De Brouwere, 2012; Witter *et al.*, 2012; Arsenault *et al.*, 2013; Ridde *et al.*, 2013; Delamou *et al.*, 2015; Ravit *et al.*, 2015; Ridde *et al.*, 2015; Boukhalfa *et al.*, 2016; Chama-Chiliba and Koch, 2016; Ganaba *et al.*, 2016; Lange *et al.*, 2016; Vallières *et al.*, 2016; Witter *et al.*, 2016; Chankham *et al.*, 2017; Edu *et al.*, 2017). Some of the costs families are bearing include food (Nahar and Costello, 1998; Khan, 2005; Witter *et al.*, 2011; Bennis and De Brouwere, 2012; Witter *et al.*, 2012; Ravit *et al.*, 2015; Boukhalfa *et al.*, 2016; Chama-Chiliba and Koch, 2016), drugs and other medical supplies (Nahar and Costello, 1998; Khan, 2005; Kruk *et al.*, 2008; Witter *et al.*, 2010; Luwei *et al.*, 2011; Arsenault *et al.*, 2013; Ridde *et al.*, 2013; Ravit *et al.*, 2015; Boukhalfa *et al.*, 2016; Lange *et al.*, 2016; Vallières *et al.*, 2016; Witter *et al.*, 2016; Chankham *et al.*, 2017), laboratory and other diagnostic tests (Nahar and Costello, 1998; Khan, 2005; Kruk *et al.*, 2008; Witter *et al.*, 2011), lodging or accommodation of the mothers or their accompanying relatives (Kruk *et al.*, 2008; Witter *et al.*, 2010; Chama-Chiliba and Koch, 2016; Witter *et al.*, 2016), transport (Nahar and Costello, 1998; Khan, 2005; Kruk *et al.*, 2008; Witter *et al.*, 2010; Bennis and De Brouwere, 2012; Arsenault *et al.*, 2013; Ravit *et al.*, 2015; Vallières *et al.*, 2016; Witter *et al.*, 2016; Edu *et al.*, 2017), either because it was not part of the policy or was part but caused significant burden, blood transfusions (Witter *et al.*, 2011; Ravit *et al.*, 2015; Lange *et al.*, 2016), wound cleaning (Witter *et al.*, 2011; Lange *et al.*, 2016), and other complications (Witter *et al.*, 2010). Other causes of OOP were referral (Bennis and De Brouwere, 2012), issuance of a child with a birth certificate (Ridde *et al.*, 2013), a family certificate to be able to access the free services (Nimpagaritse and Bertone, 2011), care of new-born (Lange *et al.*, 2016; Witter *et al.*, 2016), hiring nurse aid (Nahar and Costello, 1998), and hospital admission fee (Khan, 2005). One study estimated the opportunity cost of for temporarily ceasing work as a result of hospitalisation (Bennis and De Brouwere, 2012).

The OOP paid by the households differed based on type and complexity of delivery (Nahar and Costello, 1998; Witter *et al.*, 2007; Luwei *et al.*, 2011; Ravit *et al.*, 2015; Boukhalfa *et al.*, 2016), type of hospital (Bennis and De Brouwere, 2012; Ganaba *et al.*, 2016), distance to the hospital (Arsenault *et al.*, 2013), area or residence whether rural or urban (Ravit *et al.*, 2015), income level and education (Nahar and Costello, 1998; Khan, 2005), and the type of interviewee (Ameur *et al.*, 2012; Ridde *et al.*, 2012b, Ridde *et al.*, 2013). For instance, mothers who underwent CS and complicated delivery had more OOP than those who had a normal delivery or assisted birth (Luwei *et al.*, 2011; Boukhalfa *et al.*, 2016). Also, the average cost of giving birth was lower in the district hospital than the regional hospital as regional hospitals were specialised hospitals (Ganaba *et al.*, 2016). Mothers who were living closer to the health facility were paying less OOP than those living far (Arsenault *et al.*, 2013), and women in rural areas spent more (Ravit *et al.*, 2015). In Bangladesh, 21% of interviewed families revealed that they were spending more than half their monthly income on maternal care, while 27% reported spending 1–8 times the income (Nahar and Costello, 1998). Also, couples with better income and education were more willing to pay OOP expenditure (Nahar and Costello, 1998). There has been differing opinion between the amounts and causes of OOP expenditure between the HCWs and patients (Ameur *et al.*, 2012; Ridde *et al.*, 2012b, Ridde *et al.*, 2013) with the patients overstating the cost and HCW indicating that lack of the essential materials was due to implementation gap of the policy which created the shortages (Ameur *et al.*, 2012).

#### Catastrophic expenditure

Five studies highlighted the element of catastrophic expenditure (Witter *et al.*, 2012; Arsenault *et al.*, 2013; The World Bank, 2013; Ganle *et al.*, 2014; Dalinjong *et al.*, 2017). Catastrophic expenditure is the consequence of suffering the burden of disease by households whose OOP spending in healthcare is more than a certain threshold of household income (Ekman, 2007; Li *et al.*, 2012). Highlighted cause of catastrophic expenditure was expensive, lengthy drug therapies required particularly for eclampsia and post-partum infections (Arsenault *et al.*, 2013), and emergency blood transfusion (Arsenault *et al.*, 2013; Dalinjong *et al.*, 2017) hospitalisation cost, consultation, lab test, transport, meals during inpatient visit (Dalinjong *et al.*, 2017), and other cost related to the policy-making healthcare spending more expensive than the food (Witter *et al.*, 2012). Others were the prohibitive cost of travelling to the health care facility to seek free treatment or for free birth was making them choose to self-medicate over the visit (Ganle *et al.*, 2014). The World Bank (2013) showed that the mode of delivery determined OOP spending in childbirth (whether vaginal or CS), choice of institution (whether public or private), cultural practices, and level of the health facility.

#### The financial effect of the policy on facilities

Nine studies highlighted the financial effects free policies had on the facilities (Witter *et al.*, 2007; Witter *et al.*, 2011; Witter *et al.*, 2012; The World Bank, 2013; Chama-Chiliba and Koch, 2016; Witter *et al.*, 2016; Dalinjong *et al.*, 2017; Ensor *et al.*, 2017). Due to funding differences, facilities in Morocco had a considerable increase of healthcare budget to support the implementation of the free caesarean policy (Witter *et al.*, 2016), while in Sudan, facilities were facing a shortfall in funding, particularly remote ones (Witter *et al.*, 2012). Different reimbursement strategies incentivised the facilities differently. For instance, Zambian facilities faced higher ANC uptake due to reduced direct costs that the patients used to face before the policy, and they reported that their income that they had been using to incentivise TBAs, buying cleaning agents, and food for inpatients had been reduced by the policy (Chama-Chiliba and Koch, 2016). The reimbursement rates of the new maternal policy in Laos PDR resulted in a marginal decrease in revenue for every delivery for their hospitals although it was predicted that a future increase in demand for delivery would increase revenue for the hospital (The World Bank, 2013). Equally, while Nepal’s policy reimbursed health facilities a higher amount for CS, there was no perverse incentive for the hospitals to choose CS over normal delivery (Ensor *et al.*, 2017).

Health facilities in Ghana reported experiencing delayed reimbursement of funds meant for the free services and were thus faced with inadequate supplies and higher OOP for patients (Dalinjong *et al.*, 2017). Equally, there was a disproportionate reimbursement to facilities based on regions and complexities of deliveries (Witter *et al.*, 2007). Also, the difference in reimbursements was caused by some regions billing based on materials used rather than the government fixed rates, while others were structuring their reimbursement plans to cater for things like training of the TBAs (Witter *et al.*, 2007). Facilities reported that they had lost incomes from petty sales to women (Witter *et al.*, 2007). Despite the policy in Ethiopia, some facilities were requesting patients to pay for normal delivery or buy birth-related supplies, and others were performing emergency delivery only when there were advanced payments made (Luwei *et al.*, 2011). In Nepal, facilities received adequate money to cover the cost of essential services and even had a surplus which they were using to incentivise their staff for excellent performance and improving the outlook of the hospitals (Witter *et al.*, 2011).

#### Informal payments or tips

The review had mixed findings on informal payments from eight studies (Khan, 2005; Masiye *et al.*, 2010; Bennis and De Brouwere, 2012; Boukhalfa *et al.*, 2016; Lange *et al.*, 2016; Vallières *et al.*, 2016; Witter *et al.*, 2016; Edu *et al.*, 2017). Reasons commonly identified for a tip and informal payment were to get favours and preferential treatment (Witter *et al.*, 2016), for performing routine activities such as pushing the patient’s trolley to and from the labour/operation room by security guards, and for HCWs to shave patients before delivery/surgery, give them enemas, and get favours such as having a bucket of hot water (Khan, 2005). In other cases, especially in hospitals that had scarce resources, patients tipped the HCWs to skip long waiting queues (Vallières *et al.*, 2016). Other payments were either overtly or covertly asked from patients by HCWs without giving clear reasons (Lange *et al.*, 2016). In other cases, it was not linked to the actual care (Bennis and De Brouwere, 2012; Boukhalfa *et al.*, 2016), and in others, it existed in the form of under the table charges (Edu *et al.*, 2017).

#### Survival tactics

Eight studies highlighted the survival tactics that families were adapting to meet the OOP (Nahar and Costello, 1998; Khan, 2005; Kruk *et al.*, 2008; Bennis and De Brouwere, 2012; Witter *et al.*, 2012; Arsenault *et al.*, 2013; Witter *et al.*, 2016; Dalinjong *et al.*, 2017). Some of the methods included using savings (Witter *et al.*, 2012; Witter *et al.*, 2016; Dalinjong *et al.*, 2017), seeking help from other family members (Nahar and Costello, 1998; Khan, 2005; Witter *et al.*, 2016), handouts and loan from money lenders (Nahar and Costello, 1998; Khan, 2005; Bennis and De Brouwere, 2012; Witter *et al.*, 2012), and selling both household and personal stuff such as carpets, chicken, jewellery (Nahar and Costello, 1998; Khan, 2005; Kruk *et al.*, 2008; Bennis and De Brouwere, 2012; Dalinjong *et al.*, 2017). Besides, some were selling livestock (Nahar and Costello, 1998; Khan, 2005; Dalinjong *et al.*, 2017), others sold land, asked for an advance from employers, dug into their business capital, and sold rice or food (Nahar and Costello, 1998; Khan, 2005), or as is in Mali, sought support from the relatives living abroad and while the poor borrowed from friends and relatives (Kruk *et al.*, 2008; Witter *et al.*, 2012; Arsenault *et al.*, 2013). In Tanzania, mothers decided to cut down on their spending (Kruk *et al.*, 2008), while in Sudan, some families chose not to receive care at all, which was even more detrimental to their lives (Witter *et al.*, 2012).

#### Equity concerns from FM policies

Eleven studies showed that despite the policies being free or were subsidised to mothers, there was a difference in the benefits received based on wealth categories (Khan, 2005; Asante *et al.*, 2007; Witter *et al.*, 2007; Ameur *et al.*, 2012; Ridde *et al.*, 2012a; Arsenault *et al.*, 2013; The World Bank, 2013; Ganle *et al.*, 2014; Philibert *et al.*, 2014; Ganaba *et al.*, 2016; Witter *et al.*, 2016). The studies showed a difference in access to normal or emergency care (Ganaba *et al.*, 2016), level of exemption, whether fully or partially (Ameur *et al.*, 2012; Ridde *et al.*, 2012a), selection of type of facility, whether private or public (Witter *et al.*, 2016), and effects and amounts of OOPs on maternal care (Asante *et al.*, 2007; Witter *et al.*, 2007; Arsenault *et al.*, 2013; The World Bank, 2013) based on wealth quantiles of the mothers. Families in rural areas in Bangladesh, for instance, had more difficulty paying the extra cost than urban households (Khan, 2005). Equally, the satisfaction level with provision of services differed based on wealth level with poor women being more satisfied (Philibert *et al.*, 2014).

#### Overall expenditure of the policy and sustainability

Only four studies highlighted concepts of the overall expenditure of the policy (Witter *et al.*, 2010; Witter *et al.*, 2011; Ganaba *et al.*, 2016; Witter *et al.*, 2016). All the four studies showed that the funds allocated for the FM policies were adequately covering the cost of the essential services. Some of the policies were implemented in phases as a strategy of ensuring sustainability. Additionally, the policies reviewed were financed domestically and were potentially sustainable, mainly if they were implemented thoroughly (Witter *et al.*, 2016).

## Discussion

To the best of our understanding, this is first integrative review that has been done to analyse the effects of the FM policies on the quality and cost outcomes. The study reviewed the roles of FM policies on aspects of provision and experience of QoC following into the WHO standards for improving maternal and child health (World Health Organisation, 2016) and the cost aspects. Overall, the studies are heterogenic and uses mixed approaches to evaluate quality and costs of maternal care with all of them being in LICs and LMICs. The results have demonstrated that there are several forms of FM policies whose overall goal is to provide access to skilled birth attendance to the pregnant mothers.

There is adequate evidence to show that the policies have significantly played a role in improving the elements of provision of quality care. Our findings reveal that FM policies are associated with positive outcomes of evidence-based practices such as improved screening for key diseases such as syphilis and HIV in pregnancy, measuring vital statistics at ANC, reducing perinatal complications, and improving immunisation. The outcomes are in line with the achievement of SDG goals 3.1 and 3.2 (United Nations Development Programme, 2016; United Nations, 2017a), and previous studies have highlighted their importance (Villar *et al.*, 2001; Lassi and Bhutta, 2015; Newman Owiredu *et al.*, 2015; Singh *et al.*, 2019a). Equally, the results show that there is very little evidence of FM policies implementing actionable information systems. While previous researchers have demonstrated that properly designed information system in maternal care could incentivise the measurement of quality care, provision of a central-based reporting tool that can potentially influence organisations’ performance and satisfaction with services (The Transforming Maternity Care Vision Team *et al.*, 2010; Frøen *et al.*, 2016; Koblinsky *et al.*, 2016), it is still a gap within the FM policies that need to be strengthened. On the other hand, findings of this study suggest that majority of the FM policies have gaps in the referral system leading to poor quality outcomes. The referral systems seem to be influenced by the reimbursement strategies that are playing a role in altering the QoC especially where some hospitals may have been holding on to patients despite not being able to meet the needs. While a sound referral system is a prerequisite to proper management of complications and achievement of international quality standards, higher-level facilities may be burdened with complications and conditions that can be effectively managed at the lower level hospitals, which is a challenge of the FM policies (Akande, 2004). Studies have shown that efficient system should be one that moves patients up the referral ladders from a lower- to higher-level facilities after providing requisite care at the lower level (Kruk *et al.*, 2018; Singh *et al.*, 2019b, Daniels and Abuosi, 2020).

The experience of care by the women, who are the target of the FM policies, is significant. Our findings show that the women’s positive cumulative experience, while utilising the services under the FM policies, could encourage them to either return for subsequent delivery or impel them to seek care elsewhere. Due to the ripple effect created by good word of mouth based of good experience, others could be encouraged to seek care. FM policies are likely to successfully boost the women’s experience through excellent communication between HCWs and the mothers, which would eventually translate to the perception of respect, preservation of dignity, and feeling of sufficient emotional support (Hulton *et al.*, 2000). However, communication and emotional support are a universal issue and not just related to FM policy. Several studies have shown a litany of factors that create positive maternal care experience. For example, interpersonal skills such as respect, courtesy, and dignity, attentiveness, rational qualities more than technical skills, decisiveness are some qualities that have been listed (Hulton *et al.*, 2000; Hulton *et al.*, 2007; Sharon Morad, 2013; Karkee *et al.*, 2014; Machira and Palamuleni, 2018). Our findings show that increased utilisation and impracticable policy expectation that HCWs are faced with in FM policies are causing increased workload resulting to a breakdown of communication to the mothers leading to a perception of disrespect and abysmal emotional support. Some procedures or actions that mothers are subjected to during delivery take long to heal and may be difficult to undo (Hulton *et al.*, 2000; Lange *et al.*, 2016). Therefore, for a desirable experience of care, the mothers should understand the treatment action and feel that their concerns are adequately addressed (Hulton *et al.*, 2000). From the policies, HCWs were perceived to be pushing women to unnecessary procedures which may have been due to asymmetry of information and a possibility that they wanted to reap the benefits of the reimbursements of the polices. Participative decision-making between patients and HCWs has been shown to build trust and preserve dignity (World Health Organisation, 2013; Zirak *et al.*, 2017) hence can be implemented by the FM policies.

There are two elements: HCWs and essential resources, which are cross-cutting on provision and experience of care. Our results have shown that HCWs play a significant role; thus, their competence and motivations, although affected by several factors, allow for the provision of better routine care under the FM policies. The motivation of HCWs is multi-faceted and has been studied by several authors (Sato *et al.*, 2017; Kok *et al.*, 2018; Musinguzi *et al.*, 2018). Our review highlighted that outcome of QoC could be altered by challenges such as include unequal distribution of staff in different regions, availability of requisite infrastructure, and incentives. Equally, the review showed that the supply of essential medication and equipment to hospitals, particularly after the implementation of FM policy, is a challenge and affects QoC. Many FM policies may be lacking critical inputs before implementation; thus, when faced with an increase in the number of users especially those who had not been previously targeted, could put pressure on available resources. Some designs of the FM policies may not be catering for replenishing strategies of the resources; therefore, a collusion cause or ripple effect may result from the increased number of patients leading to poor quality of service and dissatisfaction. Other studies have also shown similar trends (El-Khoury *et al.*, 2011; Touré *et al.*, 2012).

The review revealed that TBAs still play an essential role in society, and some women prefer them to midwives in hospitals despite FM policies. TBAs have the potential to alter the intended progress envisaged by most FM policies; hence, there is a need to involve them intelligently. Some of the TBAs are retired nurses and women prefer them because they provide better personal relationships and individualised attention during birth which many hospitals lack because of the increased number of deliveries because of the free policies. Other research has shown that some pregnant mothers perceive hospital staff and services poorly hence choose to deliver through the support of TBA (Oyerinde *et al.*, 2013). While research has shown the mothers lack of resources is a contributor to TBA visits, the role of free policies should be to relieve the mothers of the payment burden.

Our review elucidated adequate evidence of the financial implications of the FM policies on patients, providers and managers, and facilities. While the goals of the policy are to improve access to skilled birth and protect mothers from financial catastrophe, the review has shown that the free policies have, in most cases, reduced family expenditure on healthcare; however, they have not eliminated OOP payment. OOP payments from patients worsened the equity gap and hindered access (Abel-Smith and Rawal, 1992; Wagstaff and Doorslaer, 2001; Ensor and Ronoh, 2005; Borghi *et al.*, 2006; Parkhurst *et al.*, 2006; Powell-Jackson *et al.*, 2010). Due to lack of intelligent monitoring systems, there are informal payments that could be eliminated by developing systems that support financial transparency and professionalism among HCWs, besides reviewing incentives. Some of the strategies have been supported in informal payment research (Lewis, 2007; Kaitelidou *et al.*, 2013; Baji *et al.*, 2015; Baji *et al.*, 2017). Also, there is a need for adequate consideration to be put to enhance sustainability of the policies especially in the design phase.

### Avenues for future research

Several studies have used different designs to show the gaps in QoC and outcomes and changes in the cost accompanying FM policies but have not shown whether the policies are the cause. Therefore, we propose that mixed-method studies or econometrics studies with intervention and control groups could be used to fully attribute the policies to changes in quality and cost of care. Also, with the debate around UHC taking shape, more political economy studies could be conducted to evaluate potential factors and designs of FM policies that can maximise on the QoC, utilisation, and cost through effective coverage measure.

### Limitations of the study

There are some limitations of the evidence from the review. For instance, our analysis was descriptive in nature, and alternate methods such as meta-analysis were not possible due to policy interventions, multiple outcomes, and the heterogeneity of studies. Also, we reviewed all studies without prior limitations of dates till 2018. There is a possibility that some studies related to our subject may have been published after 2018, but we wanted to focus on the period that captures best the discourse of initiation of UHC. Equally, some FM policies may have changed in design throughout the pilot periods or implementations, and we wanted to limit the heterogeneity.

## Conclusion

There is a gap in quality care and outcomes of the FM policies that can adequately be reduced if the design of the policies actively invests in quality as significant objective rather than just enhancing utilisation. The policies can reduce the financial burden of the households if well implemented and sustainably funded. Besides, they may also contribute to the relative decline in inequity between the rich and poor though not independently. In order to achieve the SDG and UHC goals that seek to ensure that everyone who requires healthcare is able to access quality service without suffering catastrophic expenditure, our study proposes the following (Table 3).

**Table 3.** Lessons for universal health coverage

1. HCWs should be at the centre of designing of the policies. Since their motivation is multifaceted, both intrinsic and extrinsic motivation factors could be included in the design phase of the policies. 2. Design of FM policies should incorporate a complimentary transport strategy that would reduce the overall OOP cost of delivery, particularly for the patients. 3. Properly equip the already existing hospitals and efficiently fund them to be able to provide quality and cost-efficient services. 4. Eliminate demeaning cultural practices, educate mothers, and improve access to quality care for all would eliminate socio-economic inequalities besides moving routine services nearer to potential users. 5. Have a better-targeted approach that would not only enhance fiscal sustainability but also reduce equity challenges. For free policies to achieve its intended objective, there was a need to improve supply-side readiness by increasing its management capacity. 6. Integrate the FM program into the public health system to enhance coverage and widen access; however, the operationalisation should have both the poor and the rich in mind aiming for equitable distribution. 7. To lessen the disparity in the delivery of FM services and eliminate inequalities, the policy designers should allocate resources based on need rather than on the size of the population. And the policy should push the benefit packages to the vulnerable population in the society especially the poor living in remote areas 8. Enhance awareness of the policy through a better communication channel to reach out better to the disadvantaged and more impoverished people especially within the rural communities 9. Implementation of a functional information system is imperative in integrating monitoring and evaluation system that will solve issues of referral and communication. 10. Provide professional development and education of HCWs to ensure their knowledge of the FM policy is up to date and their interpersonal skills are enhanced.

## Supplementary material

For supplementary material accompanying this paper visit http://dx.doi.org/10.1017/S1463423621000529. click here to view supplementary material

## Availability of data and material

All data sets used and or analysed during the study are available from the corresponding author on a reasonable request.
