# Supplementary material for: Effects of free maternal policies on quality and cost of care and outcomes: an integrative review
Source: Prim Health Care Res Dev. 2021 Sep 15;22:e43. doi: 10.1017/S1463423621000529 (PMC8444462; doi:10.1017/S1463423621000529)
Supplement: Supplementary file 1 [file S1463423621000529sup001.docx]

*Additional file 1: Characteristics of the studies reviewed*

| **Author (year)** | **Country (Country category)** | **Aim** | **Study Design** | **Type of Study/ method and approach** | **Peer review/ Grey literature (Source)** | **Quality of the article** |
| --- | --- | --- | --- | --- | --- | --- |
| Ameur et al. (2012) | **Burkina Faso** (LIC) | To investigate the impact of these interventions on the out-of-pocket expenditures of households for non-complicated institutional births in three rural health districts of Burkina Faso | **Case control** study | **Quantitative analysis** using data from women who gave birth in health centres and had no complications | Peer-review  (MEDLINE) | High |
| Arsenault et al (2013) | **Mali** (LIC) | To investigate the frequency of catastrophic expenditure for emergency obstetric care, explore its risk factors, and assess the effects on households | **Case control** study | **Quantitative analysis** using data on 484 emergencies (First survey utilised social autopsy interview method while Second survey there was a household survey) | Peer-review  (MEDLINE) | High |
| Asante et al. (2008) | Central and Volta region, **Ghana** (LMIC) | To evaluate the economic outcomes of the policy on households in Ghana | **Cross sectional household survey** | **Quantitative analysis** using household cost-survey data | Peer-review (MEDLINE) | Medium |
| Bennis and De Brouwere: (2012) | **Morocco** (LMIC) | To estimate the actual cost of caesarean sections from the patients’ perspective | **Cross-sectional** study | **Quantitative analysis** using data from semi-structured questionnaires with mothers who gave birth in the policy, husbands and accompanying relatives | Peer-review (MEDLINE) | Low |
| Bosu et al. (2007) | Central and Volta region, **Ghana** (LMIC) | To examine the effect of the exemption policy on delivery-related maternal mortality | **Before and after intervention study design** | **Quantitative analysis** using data obtained from all registers (Admission and discharge registers in the female wards, Outpatient, Maternity, theatre, emergency room, isolation, ICU and mortuary) | Peer-review (MEDLINE) | Medium |
| Boukhalfa et al (2016) | **Morocco** (LMIC) | To assess the policy effectiveness by analysing household expenditures related to childbirth, by delivery type and quintile | **Cross-sectional** quantitative study | **Quantitative analysis** using data from structured questionnaire with 973 women | Peer-review (MEDLINE) | High |
| Chama-Chiliba and Koch (2014) | **Zambia** (LMIC) | To examine regional differences in the effect of user fee removal in rural areas of Zambia on the use of health institutions for delivery | **Quasi experimental design** | **Econometrics analysis** using Zambia Demographic and Health Survey (ZDHS); Zambia Health Facility Census (ZHFC); Ministry of Health’s (MoH) Health Management and Information Health System (HMIS) | Working paper (Website on Health Financing) | Medium |
| Chama-Chiliba and Koch (2016) | **Zambia** (LMIC) | To analyse the effect of user fee removal on the use of public health facilities for childbirth | **Interrupted time series (ITS) design** | **Econometrics analysis** using Ministry of Health’s (MoH) of Zambia Health Management and Information Health System (HMIS) | Peer-review (MEDLINE) | Medium |
| Chankham et al. (2017) | Oudomxay Province, **Lao PDR** (LMIC) | To ascertain the knowledge level about free policy among Lao women and to determine their level of satisfaction with the maternal service provision | **Cross sectional** study | **Quantitative analysis** using data from structured questionnaire with 360 women who delivered their children at the health facilities from July 2014 to June 2015 | Peer-review (PUBMED) | High |
| Dalinjong et al. (2017) | Nothern **Ghana** (LMIC) | To estimate OOP payments and the financial impact on women during childbirth in one rural and poor area of Northern Ghana | Descriptive **convergent parallel mixed method design** | **Mixed-methods approach** using structured questionnaire for OOP payments; FGD using semi structured interview guide | Peer-review (MEDLINE) | Medium |
| Delamou et al (2015) | **Guinea** (LIC) | To assess the changes in coverage of obstetric care before and after the implementation of free emergency obstetric policy | Descriptive **cross-sectional study** | **Quantitative analysis** using retrospective review of routine program data | Peer-review (MEDLINE) | High |
| Edu et al. (2017) | Cross River State Government of **Nigeria** (LMIC) | To evaluate the effect of the free maternal health care program on the health care-seeking behaviours of pregnant women | **Observational study** | **Mixed-methods approach** using utilisation data obtained from PROJECT HOPE, CRS Ministry of Health and Nigeria DHS; Focus Group Discussions (FGD) with pregnant and postpartum mothers | Peer-review (MEDLINE) | Low |
| Ensor et al. (2017) | **Nepal** (LMIC) | To examine the impact of these mechanisms on access to safe delivery services | **Quasi experimental design** | **Econometrics analysis** using 3 rounds of 2001, 2006, and 2011 National demographic Health survey | Peer-review (PUBMED) | High |
| Ganaba et al. (2016) | **Burkina Faso** (LIC) | To examine the effects on utilisation, quality of care, equity, cost and sustainability of the subsidy policy in Burkina Faso five years after implementation | **Complex evaluation** using realist approach (Using **case study approach**) | **Mixed-methods approach** using structured questionnaire (1,609 household interviews; 130 health worker surveys); medical record extraction 1,752; key informant interviews | Peer-review (CINAHL) | High |
| Ganle et al. (2014) | **Ghana** (LMIC) | To explore health system factors that inhibit women’s access to and use of skilled maternal and newborn healthcare services in Ghana despite these services being provided free | **Qualitative study** design | **Qualitative analysis** using focused group discussion and Key informant interviews | Peer-review (MEDLINE) | High |
| Khan (2005) | **Bangladesh** (LMIC) | To investigate the out-of-pocket expenditures by patients for free services in a large public hospital in Bangladesh, factors influencing expenses, and their impact on household income. | **Cross sectional survey** | **Quantitative analysis** using semi-structured in-depth interviews with 81 mothers | Peer-review (Hand search of references from identified literature) | Low |
| Koroma et al. (2017) | Bomali District, **Sierra Leone** (LIC) | To investigate the quality of antenatal and delivery services provided in health facilities implementing the FHCI and to identify solutions to overcome identified barriers that are preventing the FHCI from being delivered effectively within districts | **Cross sectional survey design** | **Quantitative analysis** using data from observations and checklist, exit interview, and review of records (obtained using WHO safe motherhood questionnaire; ANC observation checklist by USAID Maternal and Child Health Integrated program) | Peer-review (MEDLINE) | Medium |
| Kruk et al. (2008) | United Republic of **Tanzania** (LMIC) | To identify the main drivers of cost for facility delivery and the financial consequences on households in a population-based sample of women in rural Tanzania | Descriptive **cross-sectional** study | **Quantitative analysis** using household survey of women who gave birth within five years | Peer-review (PUBMED) | medium |
| Lange et al. (2016) | **Benin** (LIC) | To explore how the Caesarean Section policy shaped health workers' and patients' perceptions of and experiences with quality of care | **Qualitative ethnographic** study | **Qualitative analysis** using observation in maternity ward, daily informal conversations, and semi structured interviews, Ethnography | Peer-review (ScienceDirect) | Medium |
| Luwei et al (2011) | **Ethiopia** (LIC) | To examine user fees for maternity services and how they relate to provision, quality, and use of maternity services in Ethiopia | Not Indicated | **Quantitative analysis** using national assessment of emergency obstetric and newborn care (EmONC) examined user fees for maternity services in 751 health facilities that provided childbirth services | Peer-review (ScienceDirect) | Low |
| Masiye et al. (2010) | **Zambia** (LMIC) | To review the performance of free health care in Zambia following 15 months of implementation | **Case study** (for qualitative part); and **before and after design** for quantitative approach | **Mixed-methods approach** using Data from Zambia Health Management and Information Health System (HMIS); Structured questionnaires for care givers; and in-depth interviews with health workers | Peer-review (ScienceDirect) | High |
| Ministry of Health, Kenya, (2015) | **Kenya** (LMIC) | To assess the implementation of free maternity services program and its effects on health service delivery | **Monitoring and evaluation** using mixed methods | **Mixed-methods approach** using qualitative interviews; KII, FGDs and exit questionnaires | A comprehensive assessment report (Personal Communication) | Low |
| Nabyonga-Orem (2008) | **Uganda** (LIC) | To prospectively study how different aspects of quality of care change, as a country changes its health financing options from user charges to free services, in a developing country setting. | Population longitudinal **cohort study** | **Mixed-method approach** using Key informant Interviews with health facility officials, FGDs with 12 Participants | Peer-review (CINAHL) | medium |
| Nahar and Costello (1998) | **Bangladesh** (LMIC) | To examine the actual costs incurred by families, and their affordability during maternity care in four government hospitals in Dhaka | **Cross sectional** survey | **Quantitative analysis** using data from structured questionnaire with 220 post-partum women | Peer-review (Hand search of references from identified literature) | Low |
| Nimpagaritse and Bertone (2011) | **Burundi** (LIC) | To draw lessons from a case study presented using service utilization from eight health providers in a district and testimonies from insiders. | Descriptive **case study** | **Mixed method approach** using direct observation; routine utilisation data and qualitative information | Peer-review (EconLit) | High |
| Philibert et al (2014) | **Burkina Faso** (LIC) | To assess whether women’s satisfaction with delivery care is maintained with a total fee exemption in Burkina Faso | **Quasi experimental design** which **case control study** | **Quantitative analysis** using data from intervention and control groups obtained from structured questionnaire on postpartum views and opinion | Peer-review (MEDLINE) | Medium |
| Ravit et al. (2015) | **Kayes Region, Mali** (LIC) | To assess any expenses that were associated with a caesarean episode in the context of user fees exemption | **Case control** approach | **Quantitative analysis** using data from structured questionnaire with 484 women, who were both maternal deaths and near-misses and experienced a caesarean section in the latter case–control study. | Peer-review (SpringerLink) | High |
| Ridde and Diarra (2009) | **Niger** (LIC) | To analyse the implementation the abolition of payment for all services for children under five years and pregnant women from the specific perspective of the actors, using an anthropological approach. | **Process Evaluation** | **Mixed-methods approach** using Individual in-depth interviews; FGDs; Participant observation; and self-administered structured questionnaire | Peer-review (MEDLINE) | Medium |
| Ridde et al. (2012) | Multi-country (**Benin** (LIC), **Burkina Faso** (LIC), **Mali** (LIC), **Niger** (LIC), **Togo** (LIC), **Senegal** (LIC)**)** | To present a transversal analysis of the results of a knowledge aggregation process undertaken with street-level bureaucrats regarding user fees exemption policies in six West African countries (Benin, Burkina Faso, Mali, Niger, Togo and Senegal) | Multiple **cases studies** with embedded level of analysis | **Mixed method approach** using self-administered questionnaire; group discussions; country team workshops | Peer-review (PUBMED) | High |
| Ridde et al. (2012) | **Burkina Faso** (LIC) | To evaluate the effects of the national maternal healthcare subsidy policy | **Cross-sectional** household survey | **Quantitative analysis** using household survey data | Peer-review (MEDLINE) | Medium |
| Ridde et al. (2015) | **Burkina Faso** (LIC) | To estimate changes in OOP spending across socio-economic strata given changes in service utilisation produced by the policy. | Repeated **cross-sectional** household survey | **Quantitative analysis** using 6 rounds of repeated household survey conducted in Part of Nouna Health District (NHD) | Peer-review (SpringerLink) | High |
| Ridde et.al (2013) | **Burkina Faso** (LIC) | To test the hypothesis that the amounts paid by women in cases of normal deliveries in primary care maternity units in Burkina Faso were more than the official fee, i.e., their 20% portion of the total cost, and if confirmed, to understand these results by means of a qualitative approach. | Descriptive and analytical implementation evaluation using **a mixed method sequential explanatory design** | **Mixed-method approach** using both qualitative and quantitative data Household survey, FGDs with the women | Peer-review (ScienceDirect) | High |
| Sidze et al. (2016) | **Kenya** (LMIC) | To investigate the impact of the FMS program on quality of maternity care in public health facilities in Kenya | Not indicated | **Mixed-methods approach** using Kenyan Demographic and Health Survey (KDHS) (2003, 2008/2009 and 2014); Health Monitoring and Information system (HMIS) secondary data on health facilities’ indicators (2011/12; 2012/13, and 2013/14); and Quantitative Survey (Exit interview questionnaire) | Grey literature (Hand search of references from identified literature) | Low |
| Steinhardt et al. (2011) | **Afghanistan** (LIC) | To synthesize lessons about the effects of user fee removal on quality—both observed facility structural quality and overall perceived quality of care—and utilization. | **Outcome Evaluation** | **Mixed-methods approach** using baseline survey; follow up facility assessment; exit interviews; household surveys; HMIS database | Peer-review (MEDLINE) | High |
| The World Bank (2013) | **Lao PDR** (LMIC) | To document the maternal OOP expenditure and service readiness | **Cross sectional** household survey | **Quantitative analysis** using household and health centre survey conducted in Lao PDR | Report (Websites on Health Financing) | Medium |
| Vallières et al (2016) | **Sierra Leone** (LIC) | To identify how the free health care initiative (FHCI) policy impacts on health inequities in Sierra Leone and how this translates into practice and interventions at the community-level. | **Cross sectional** household survey | **Quantitative analysis** using baseline evaluation data for MCH program implemented in Bonthe District | Peer-review (MEDLINE) | Low |
| Witter et al (2010) | **Senegal** (LIC) | To provide evidence of effectiveness of free policy in Senegal and recommend ways to improve its implementation | **Policy evaluation** | **Mixed-methods approach** using semi-structured interviews with key informant; forms for extraction of financial information; Unstructured discussion at the community level; Structured questionnaire applied to clinical records | Peer-review (MEDLINE) | High |
| Witter et al. (2007) | **Ghana** (LMIC) | To describes Ghana's innovative scheme that exempt all women from delivery fees | Policy **baseline evaluation** | **Qualitative analysis** using key informant and semi structured interviews | Peer-review (MEDLINE) | High |
| Witter et al. (2013) | **Ghana** (LMIC) | To explore the policy process and the early implementation of Ghana’s free NHIS policy for pregnant women | An exploratory **process evaluation** | **Mixed-methods approach** review of literature both grey and published and key informant interviews with stakeholders | Peer-review (MEDLINE) | High |
| Witter et al. (2011) | **Nepal** (LMIC) | To understand the effects of the free policy on health facilities | Monitoring and evaluation using a **before and after study design** | **Mixed-methods approach** using reviewed district records; Structured extraction from facility data; and Semi-structured interviews | Peer-review (MEDLINE) | High |
| Witter et al. (2012) | **Sudan** (LMIC) | To document implementation challenges and effectiveness of fee exemption policy in Sudan | Policy **implementation evaluation** | **Mixed-methods approach** using semi-structured interviews with key informant; forms for extraction of financial information; Structured questionnaire applied to clinical records | Peer-review (MEDLINE) | High |
| Witter et al. (2016) | Multi-country (**Benin** (LIC), **Burkina Faso** (LIC), **Mali** (LIC), **Morocco** (LMIC)) | To document the costs and impacts of obstetric fee removal and reduction policies in a holistic way | Comparative **case study design** based on realist evaluation | **Mixed-methods approach** using FEMHealth research tools (observation grid, interview guide with actors, policy documents, financial flow tracking, Costing interviews, Exit interviews, Health worker survey policy implementation assessment, policy effect mapping study, Realist case study, quantitative instrument on near-miss, CS and quality, Quantitative analysis of secondary data | Peer-review (CINAHL) | High |
| Witter et al. (2017) | Multi-country (**Benin** (LIC), **Burkina Faso** (LIC), **Mali** (LIC), **Morocco** (LMIC)) | To evaluate the costs and effectiveness of national obstetric fee exemption policies | Comparative **case study design** based on realist evaluation | **Mixed-methods approach** using Document review; Key Informant Interviews; Secondary data; Structured extraction from medical files; Observation of care processes. Questionnaire was developed and tested in Ghana | Peer-review (MEDLINE) | High |
